# Use of 18F-FDG PET/CT Imaging for Radiotherapy Target Volume Delineation after Induction Chemotherapy and for Prognosis of Locally Advanced Squamous Cell Carcinoma of the Head and Neck

**DOI:** 10.3390/medicina54060107

**Published:** 2018-12-10

**Authors:** Viktoras Rudžianskas, Erika Korobeinikova, Milda Rudžianskienė, Evelina Jaselskė, Diana Adlienė, Severina Šedienė, Ilona Kulakienė, Evaldas Padervinskis, Nemira Jurkienė, Elona Juozaitytė

**Affiliations:** 1 Oncology Institute of Lithuanian University of Health Sciences, Eivenių g. 2, 50009 Kaunas, Lithuania; viktoras.rudzianskas@lsmuni.lt (V.R.); milda.rudzianskiene@lsmuni.lt (M.R.); severina.sediene@lsmuni.lt (S.Š.); kulakiene@dr.com (I.K.); evaldas.padervinskis@lsmuni.lt (E.P.); nemira.jurkiene@lsmuni.lt (N.J.); elona.juozaityte@lsmuni.lt (E.J.); 2 Faculty of Mathematics and Natural Sciences, Department of Physics, Kaunas University of Technology, Studentų g. 50, 51368 Kaunas, Lithuania; evelina.jaselske@ktu.edu (E.J.); diana.adliene@ktu.lt (D.A.)

**Keywords:** head and neck cancer, induction chemotherapy, ^18^F-FDG PET/CT, target volume delineation

## Abstract

*Background and objectives:* Induction chemotherapy (ICT) before definitive chemoradiation (CRT) gives high response rates in locally advanced squamous cell carcinoma of the head and neck (LA-SCCHN). However, pre-ICT gross tumor volume (GTV) for radiotherapy (RT) planning is still recommended. As ^18^F-FDG PET/CT has an advantage of biological tumor information comparing to standard imaging methods, we aimed to evaluate the feasibility of ^18^F-FDG PET/CT-based post-ICT GTV delineation for RT planning in LA-SCCHN and to assess the prognostic value of PET parameters: maximum standardized uptake value (SUV_max_), metabolic tumor volume (MTV) and total lesion glycolysis (TLG). *Methods:* 47 LA-SCCHN patients were treated with 3 cycles of ICT (docetaxel, cisplatin, and 5-fluorouracil) followed by CRT (70 Gy in 35 fractions with weekly cisplatin). Pre- and post-ICT PET/CT examinations were acquired. Planning CT was co-registered with post-ICT PET/CT and RT target volumes were contoured according to post-ICT PET. Post-ICT percentage decrease of SUV_max_, MTV and TLG in primary tumor and metastatic regional lymphnodes (LN) was counted. Loco-regional failure patterns, 3-year progression free (PFS) and overall survival (OS) were evaluated. *Results:* 3-year PFS and OS rates for study population were 67% and 61% respectively. 31.9% of patients progressed loco-regionally. All progress was localized in high-to-intermediate dose (60–70 Gy) RT volumes and none in low dose (50 Gy) volumes. Decrease of SUV_max_ ≥ 74% (*p* = 0.04), MTV ≥ 68% (*p* = 0.03), TLG ≥ 76% (*p* = 0.03) in primary tumor, and LN TLG decrease ≥ 74% (*p* = 0.03) were associated with PFS. Decrease of primary tumor SUV_max_ ≥ 74% (*p* = 0.04), MTV ≥ 69% (*p* = 0.03), TLG ≥ 74% (*p* = 0.02) and LN TLG ≥ 73% (*p* = 0.02) were prognostic factors for OS. *Conclusions:* According to our results, ^18^F-FDG PET/CT-based post-ICT GTV delineation is feasible strategy without negative impacts on loco-regional control and survival. Percentage decrease of metabolic PET parameters SUV_max_, MTV and TLG has a prognostic value in LA-SCCHN.

## 1. Introduction

Despite the absence of definitive scientific evidence, induction chemotherapy (ICT) for locally advanced squamous cell carcinoma of the head and neck (LA-SCCHN) is often used in clinical practice [1]. Up to 80–90% of patients with LA-SCCHN respond to cisplatin-based ICT and 20–40% of them achieve complete response (CR) [2]. Although several studies reported no benefit of ICT in terms of survival [2,3,4], it has a role in selected cases if there is likely to be a delay between diagnosis and starting definitive chemoradiotherapy (CRT) and in organ preservation strategies [5].

The current guidelines suggest that pre-ICT primary site and nodal gross tumor volumes (GTV) should be used for radiotherapy (RT) planning in cases when ICT is given [6,7,8]. However, possible superiority of post-ICT over pre-ICT GTV is now under investigation [9,10]. The potential advantages of using post-ICT imaging for target volume delineation include the reduction of GTV due to tumor shrinkage and the possibility to spare normal tissues. However, it bears a potential risk of missing partial tumor volume and, in some cases, difficulties in GTV delineation due to metabolic switch [9,11].

Contrast-enhanced computed tomography and/or magnetic resonance imaging are the standard methods for evaluating tumor response to ICT, and are mostly used for GTV delineation in LA-SCCHN. Recent studies report an emerging role of positron emission tomography (PET) with ^18^Fluorine-labeled 2-fluoro-2-deoxyglucose integrated with computed tomography (^18^F-FDG PET/CT) in RT planning for head and neck cancers due to the added biological tumor information. However, there is a lack of knowledge regarding whether the use of ^18^F-FDG PET/CT based post-ICT GTV delineation in RT planning is a feasible approach.

Furthermore, earlier clinical studies demonstrated, that some parameters of ^18^F-FDG PET/CT may predict the tumor chemosensitivity and LA-SCCHN patient survival [12,13,14]. Such ^18^F-FDG PET/CT parameters as maximum standard uptake value (SUV_max_), metabolic tumor volume (MTV) and total lesion glycolysis (TLG) have been shown to correlate with LA-SCCHN patient outcome [15,16,17]. However, the prognostic value of the percentage decrease of these FDG uptake parameters from baseline to post-ICT is unknown.

The aims of our prospective phase II study were:

1. To evaluate the feasibility of ^18^F-FDG PET/CT-based post-ICT GTV delineation strategy for LA-SCCHN RT planning by analyzing patterns of local and/or nodal disease failure after CRT and assessing progression free survival (PFS) overall survival (OS) and treatment safety;
2. To assess the correlation of post-ICT percentage decrease of three metabolic^18^F-FDG PET/CT parameters SUV_max_, MTV and TLG in primary tumor and metastatic nodes with the radiological response to ICT, PFS and OS.

## 2. Material and Methods

### 2.1. Study Design and Patient Selection

Between September 2013 and January 2016, patients affected by LA-SCCHN were enrolled in this phase 2 prospective cohort study. All subjects signed the informed consent form. The study was conducted in accordance with the Declaration of Helsinki, and the protocol was approved by the Kaunas Regional Ethics Committee for Biomedical Research (No. BE-2-51; 5 November 2013) and was registered at www.clinicaltrials.gov (identification no. NCT02047201). All patients have been validated by the multidisciplinary team for ICT followed by CRT. The eligibility criteria were as follows: histologically confirmed locally advanced (stage III and IV) head and neck squamous cell carcinoma, Eastern Cooperative Oncology Group performance status (ECOG) 0 or 1, signed written informed consent. Patients with a known history of another cancer or suspected metastatic lesions were excluded.

### 2.2. Induction Chemotherapy Delivery and Evaluation of Response to ICT

Patients received three cycles of docetaxel, cisplatin, and 5-FU (TPF) ICT consisting of docetaxel (75 mg/m^2^) and cisplatin (75 mg/m^2^) administered as a 1-h infusion on a day 1 and 5-fluorouracil (5-FU) (750 mg/m^2^) administered by continuous infusion on days 1–5 [18]. Cycles were administered every 3-weeks. The reductions of the docetaxel, cisplatin and 5-FU doses were planned depending on the individual treatment tolerance and toxicity.

According to post-ICT ^18^F-FDG PET, patients were classified into: ICT-responders—patients with ≥50% visual tumor volume reduction, and ICT-non-responders—<50% reduction [19,20].

### 2.3. ^18^F-FDG PET/CT Examination and Gross Tumor Volume Delineation

Two ^18^F-FDG PET/CT examinations were performed for each patient, both consisting of whole-body scan and localized high-resolution head and neck scan. The first imaging was accomplished prior ICT during initial staging, another one occurred 14 ± 2 days after the last cycle of ICT for therapeutic evaluation and CRT planning [7,11,13]. Patient’s preparation for the scan included a low carbohydrate diet for at least 24 h prior the scan and fasting for more than 6 h. Serum glucose level was measured on the day of the scan and aimed to be <7 mmol/L. The injected activity of ^18^F-FDG was 4 MBq/kg of body weight. After injection patients remained in a quiet room for approximately 60 min. A whole-body PET/CT scan was acquired from the skull base to the mid-thigh with the patient’s hands above the head. The examination was started with low-dose CT (120 kV, 100 mA, 3.75 mm section thickness) followed by PET acquisition (3 min per bed position). Localized high resolution head and neck PET/CT scan was acquired with the patient positioned with arms along the body. PET acquisition allowed 5 min per bed position. In addition, post-ICT head and neck scan was performed while patients were positioned on a radiation therapy planning table in intensity modulated radiation therapy (IMRT) treatment position with the five-point fixation thermoplastic mask. All ^18^F-FDGPET/CT studies were performed on GE Discovery XCT (General Electric Healthcare system, United States of America) scanner. All PET/CT images were reconstructed with the ordered subset expectation maximization (OSEM) iterative algorithm using scatter correction with the 5 mm Gaussian filter on 128 × 128 and 256 × 256 matrixes.

All acquired data was transferred to radiotherapy treatment planning system Eclipse (version v8.6) and registered by aligning the centers of the datasets. Planning CT was rigidly co-registered with post-ICT ^18^F-FDGPET/CT for delineation of treatment volumes. The gross tumor volume GTV70 and gross nodal volume GTV60 were manually contoured using a visual interpretation technique on post-ICT PET images by radiation oncologist in collaboration with an experienced nuclear medicine physician. Clinical target volumes (CTV) of primary tumor CTV70 obtained by adding 5 mm margin to GTV70. CTV60 involved GTV70 and GTV60 plus 5 mm margin. The elective CTV (CTV50) included CTV70, CTV60 and bilateral elective lymph nodes. The margin of 3 mm was added for each CTV to create the planning target volumes (PTV) PTV70, PTV60 and PTV50 (during RT delivery daily cone-beam CT (CBCT) for image guidance was used). For high-risk volumes PTV70 and PTV60 the prescribed doses were 70 Gy and 60 Gy respectively, for PTV50—50 Gy.

Three metabolic parameters (SUV_max_, MTV and TLG) were measured in pre-ICT and post-ICT ^18^F-FDGPET/CT scans for both primary tumor and metastatic regional lymphnodes. Around every suspicious lesion, the isocontour region of interest centered on the maximum value pixel was drawn automatically by workstation tools (Metavol software v. 1.4.) generating SUV_max_ and mean SUV (SUVmean) of the region. Metabolic tumor volume was drawn manually contouring margin of every lesion and then segmented semi-automatically in three dimensions. MTV was defined as the summed volume in cubic centimeters (cm^3^). TLG was calculated as the product of MTV and SUVmean. 

### 2.4. Chemoradiotherapy

CRT consisted of cisplatin 40 mg/m² weekly concomitant with conventionally fractionated RT (2 Gy per once-daily fraction, 5 days a week until the total prescribed dose of 70 Gy will be collected) [21,22]. The cisplatin dose was individually modified according to the level of hematologic toxicity, hepatic and renal function and the infectious diseases. Non-completion of prescribed radiotherapy dose was related to individual patient tolerance.

### 2.5. End Points

PFS and OS were used as the clinical endpoints to evaluate the feasibility of ^18^F-FDG PET/CT-based post-ICT GTV delineation and the prognostic value of the ^18^F-FDG PET metabolic parameters. PFS was defined as the *time* from *day 1* of the *ICT first cycle* until disease progression or death from any reason. OS was defined as the period from *day 1* of the *ICT first cycle* until death from any reason. Furthermore, to evaluate the safety of new target volume delineation technique, treatment toxicity during ICT and CRT was evaluated on a weekly basis according to the National Cancer Institute Common Toxicity Criteria (NCI CTCAE) v.4.0. Late adverse events related with RT were assessed every three months after CRT using RTOG (Radiation Therapy Oncology Group)/EORT (European Organization for Research and Treatment of Cancer) toxicity criteria.

### 2.6. Analysis of Loco-Regional Failure Patterns

Diagnostic CT or PET/CT documenting recurrence was co-registered with planning CT and pre-ICT PET/CT using rigid image registration technique. Alternative IMRT treatment plans basing on pre-ICT PET/CT were also created to investigate the relationship between failure site and primary PET/CT visual tumor volume. Loco-regional failures were classified depending on the percentage volume which received in total 95% of the prescribed dose: failures were classified as “in-field” (>95%), “marginal” (≥20%; ≤95%) and “out-field” (<20%) [23,24]. Mean, minimum and maximum dose and D95 to the failure volume were estimated. 

### 2.7. Statistics

Means of pre-ICT, post-ICT and the percentage decrease of SUV_max_, MTV and TLG were compared between ICT-responder versus ICT-non-responder groups using Student’s *t*-test. ROC curve analysis was applied to identify the best discriminating cut-off values for SUV_max_, MTV, and TLG to predict PFS and OS. Appropriate cut-off was defined as the point on the curve nearest to the upper left corner of the receiver operating characteristics (ROC) graph. The area under the curve (AUC) was used to evaluate the accuracy of the metabolic parameters as a prognostic factor. The Kaplan—Meier method was applied to estimate PFS and OS. The Cox regression model was applied for the estimation of the hazard ratio (HR); and for the multivariate analysis using a forward selection. Log-rank test (Mantel–Cox) was used to compare survival distributions. All tests were two-sided, and the significance threshold was set at *p* < 0.05. All statistical analyses were performed using IBM SPSS 22.0 (Statistical Package for Social Sciences 22.0 for Windows) statistical software.

## 3. Results

### 3.1. Patient Characteristics

**Figure 1:**
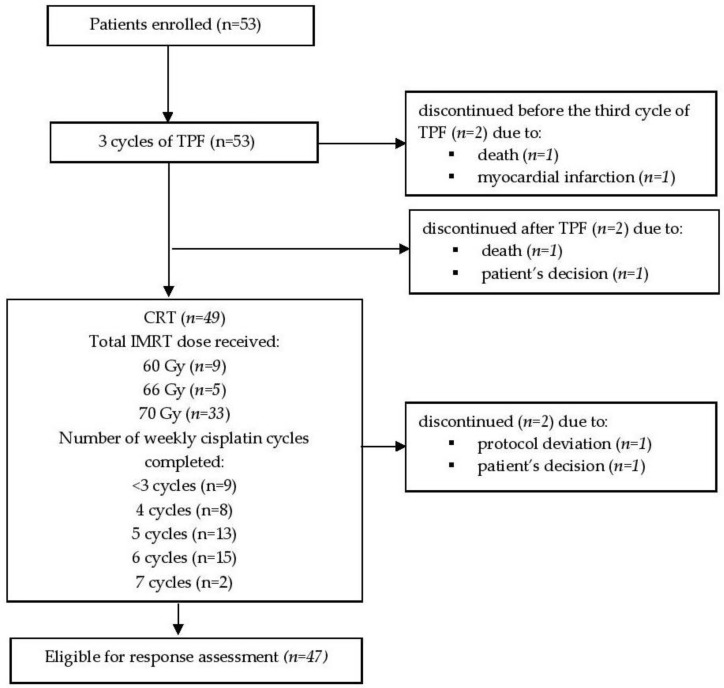
Flow diagram of study participants. TPF—induction chemotherapy consisting of docetaxel, cisplatin, and 5-fluorouracil (5-FU); IMRT—intensity modulated radiation therapy; CRT—chemoradiotherapy.

In total 53 patients (50 males and 3 females) with mean age at diagnosis of 55.8 ± 8.9 (range 30–71) years were enrolled in this study. As demonstrated in flow diagram of study participants (Figure 1), 47 patients completed ICT and CRT and were eligible for further analysis. 21 (44.7%) of analyzed patients had primary hypopharyngeal carcinoma and 26 (55.3%)—oropharyngeal carcinoma (all cases negative for human papillomavirus). Patient characteristics at the time of diagnosis are listed in Table 1.

### 3.2. Clinical Outcome of 18F-FDG PET/CT-Based Post-ICT GTV Delineation Strategy

**Figure 2:**
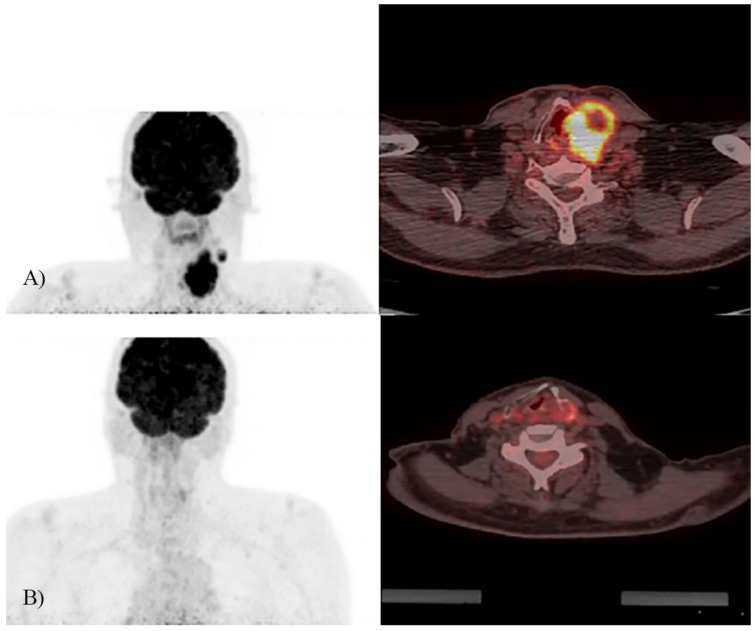
A representative LA-SCCHN case of good response (>50% visual tumor volume reduction) to induction chemotherapy. (**A**) Pre-ICT 18F-FDG PET/CT; (**B**) post-ICT 18F-FDG PET/CT of the same patients with cT4N1M0 squamous cell carcinoma of the left pyriform sinus.

**Figure 3:**
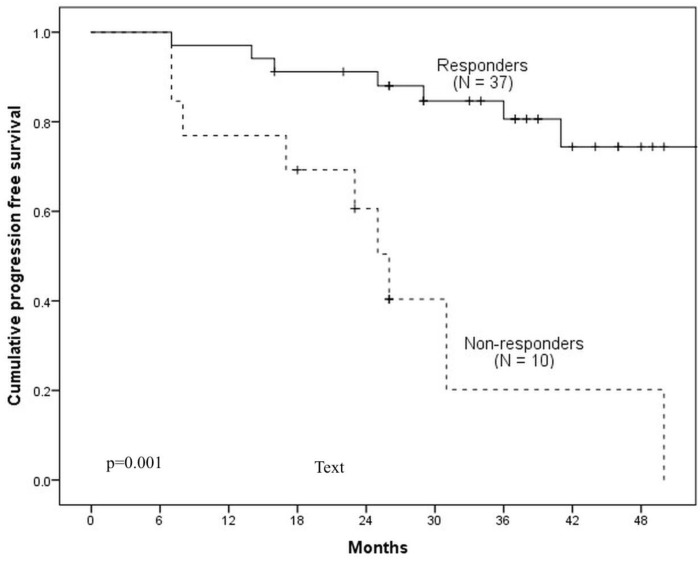
Comparison of PFS between ICT-responders and non-responders.

**Figure 4:**
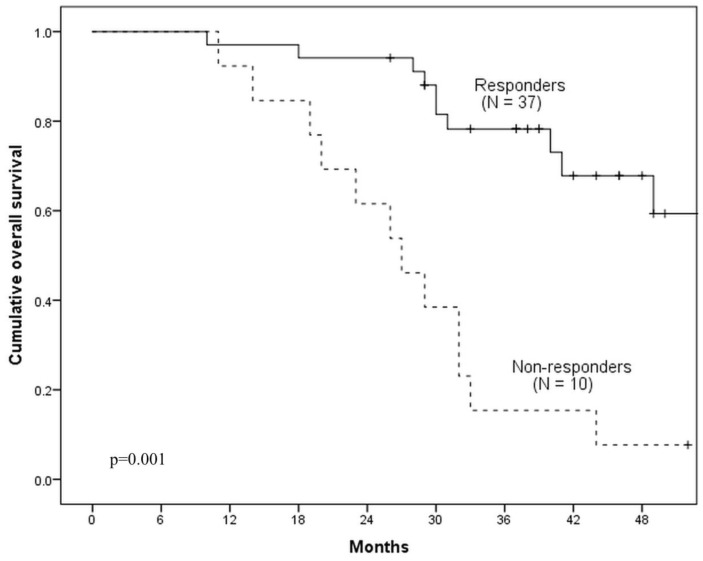
Comparison of OS between ICT-responders and non-responders.

Median follow-up of patients was 36.4 months (range: 9.7–57.2). 3-year PFS and OS rates for all study population were 67% and 61% respectively. According to post-ICT ^18^F-FDG PET/CT, 10 (21.3%) patients were ICT-non-responders and 37 (78.7%)—ICT-responders (a representative case of ICT-responder is demonstrated in Figure 2). The 3-year PFS for ICT-non-responders was 20%, comparing with 84% for ICT-responders (Hazard ratio (HR) = 6.5, 95% CI 2.33–18.7; *p* = 0.001) (Figure 3). The 3-year OS for ICT-non-responders was 16% vs. 78% for ICT-responders (HR = 5.47, 95% CI 2.33–12.83; *p* = 0.001) (Figure 4). The median PFS and OS were not reached.

### 3.3. Patterns of Loco-Regional Failures

Loco-regional disease failure occurred in 15 patients (31.9%) (7 in ICT-responder group and 8 in ICT-non-responder group). For 5 patients (10.6%) it was allocated at the primary tumor site, 7 patients (14.9%) had local control but regional failure and 3 patients (6.4%) had both primary and nodal failure. Every single recurrent lesion was analyzed separately and in total 32 locoregional recurrent lesions of 15 patients were analyzed. Relative to the percentage target volume that received 95% of the prescribed dose for specific PTV, a total of 19 (59.3%) failures were classified as in-field, 7 (21.9%) failures were defined as marginal and 6 (18.8%) failures as out-field. The average mean (± standard deviation (SD)), minimum, maximum dose and *dose* to 95% of planning target volume (D95) did not differ significantly among in-field, marginal and out-field recurrence sites (Table 2).

The primary tumor persisted or recurred at the site of high-dose GTV (GTV70) and high-dose CTV (CTV70) in 7 (87.5%) cases, and 1 (12.5%) case occurred in high-dose PTV (PTV70). The failure of LN observed in intermediate-dose GTV (GTV60) and intermediate-dose CTV (CTV60) in 21 (87.5%) cases, and 3 (12.5%) cases in intermediate-dose PTV (PTV60). No failures were observed in prophylactic dose PTV (PTV50). In high dose PTV70 volumes, significantly lower dose coverage was observed (Table 2).

Furthermore, patterns of locoregional failure in alternative virtual IMRT plans based on pre-ICT PET/CT images were analyzed. Primary tumor site failures were classified as in-field in 6 (75%) cases, marginal in 2 (25%) cases and none as out-field. As for LN failures: 18 (75%) in-field, 4 (16.7%) marginal and 2 (8.3%) out-field. Evaluating radiation target volumes, 6 recurrences of primary tumor were in GTV70 and 2 in CTV70. The failure of LN recurred at the site of intermediate-dose GTV60 in 18 cases, 3 cases in CTV60 and 3 cases in PTV60. No failures were observed in prophylactic dose PTV (PTV50). In intermediate-dose PTV60 volumes, significantly lower PTV dose coverage was observed (Table 3). There were no significant differences between pre-ICT and post-ICT IMRT plans according to failure localization, D95, mean, minimum and maximum dose.

### 3.4. Toxicities

During CRT the rates of grade 3–4 dermatitis, mucositis, anemia, leukopenia and thrombocytopenia were 27.6%, 21.2%, 6.4%, 6.4% and 6.4%, respectively. No grade 3 or 4 xerostomia or body-weight loss were observed. The incidence of grade 3–4 late toxicities was: 1 (2.1%) patient developed osteoradionecrosis, 1 (2.1%) developed trismus, 2 (4.3%) dysphagia and 1 (2.1%) bleeding.

### 3.5. ROC Curve Analysis, AUC and Cut-Off Values of the Metabolic Parameters

The mean of SUV_max_ percentage decrease of the primary lesions after ICT was 58.9 ± 24.7% and the mean of MTV and TLG percentage decrease of the primary lesions were 63.4 ± 33.6% and 66.3 ± 41.2% respectively. The mean of SUV_max_, MTV and TLG percentage decrease of metastatic LN were 38.5 ± 32.8%, 38.5 ± 32.8% and 64.9 ± 34.6% respectively. The abilities of the SUV_max_, MTV, and TLG post-ICT percentage decrease values to predict PFS and OS were calculated by ROC curves. AUC of the tested parameters, their optimal cut-off values for PFS and OS are demonstrated in Table 4.

### 3.6. Prognostic Value of the Metabolic Parameters

Associations of three metabolic ^18^F-FDG PET/CT parameters SUV_max_, MTV and TLG in primary tumor and metastatic nodes with post-ICT tumor volume reduction are demonstrated in the Table 5 and Table 6. Tumor response was associated with primary tumor parameters: pre-ICT MTV (*p* < 0.001) and TLG (*p* = 0.02); post-ICT SUV_max_ (*p* = 0.004), MTV (*p* < 0.001) and TLG (*p* < 0.001); percentage decrease of SUV_max_ (*p* = 0.027) and MTV (*p* = 0.001). Tumor response was also statistically significantly linked with metastatic LN parameters: pre-ICT MTV (*p* < 0.001) and TLG (*p* < 0.001); post-ICT MTV (*p* = 0.006) and TLG (*p* < 0.001). Percentage decrease of LN metabolic parameters was not associated with tumor response.

According to the univariate Cox regression analysis (Table 7 and Table 8), percentage decrease of primary tumor SUV_max_ ≥ 74%, MTV ≥ 68%, TLG ≥ 76%, metastatic LN SUV_max_ ≥ 68% and TLG ≥ 74% and lymphnode status N were significant prognostic factors for PFS. Percentage decrease of primary tumour SUV_max_ ≥ 74%, MTV ≥ 69%, TLG ≥ 74%, metastatic LN SUV_max_ ≥ 69% and TLG ≥ 73% and lymphnode status N were significant prognostic factors for OS.

In multivariate analysis (Table 7 and Table 8) primary tumor SUV_max_ decrease ≥ 74% (HR = 1.4; 95% CI 1.2–9.3; *p* = 0.04), primary tumor MTV decrease ≥68% (HR = 1.9; 95% CI 1.3–11.5; *p* = 0.03), primary tumor TLG decrease ≥ 76% (HR = 3.7; 95% CI 1.2–9.3; *p* = 0.03), LN TLG decrease ≥ 74% (HR = 2.9; 95% CI 1.3–6.9; *p* = 0.03) and N stage (HR = 2.8; 95% CI 1.7–16.3; *p* = 0.02) remained significantly associated with PFS. Moreover, ≥74% decrease in primary tumor SUV_max_ (HR = 1.2; 95% CI 1.1–5.8; *p* = 0.04), ≥69% decrease in primary tumor MTV (HR = 1.8; 95% CI 1.3–6.7; *p* = 0.03), ≥74% decrease in primary tumor TLG (HR = 3.1; 95% CI 1.4–17.6; *p* = 0.02), ≥73% decrease in LN TLG (HR = 2.6; 95% CI 1.4–6.2; *p* = 0.02) and N stage (HR = 2.2; 95% CI 1.4–8.7; *p* = 0.02) remained independent prognostic factors for OS.

## 4. Discussion

Our study is the first to investigate the feasibility of target volume delineation based on post-ICT ^18^F-FDG PET/CT images for LA-SCCHN IMRT planning. The rationale for this approach is the potential reduction of GTV after ICT leading to mitigation of RT toxicities by sparing normal tissues. Several authors in their publications have discussed post-ICT imaging-based target volume delineation strategy [6,8,9,25]. However, due to the lack of supportive data they still recommend the use of pre-induction primary tumour and nodal GTVs but further clinical research is needed.

Survival data from our study cannot be directly compared with the results of other authors because there are no completed clinical trials analysing post-ICT PET/CT based RT planning. For indirect comparison patients’ outcomes of several clinical studies, where ICT plus CRT approach was used for LA-SCCHN treatment are presented in Table 9. One of the earliest published studies analysing LA-SCCHN patients treated with ICT (TPF) plus CRT was TAX-324 [26]. In this study 3-year PFS and OS rates were 50% and 62% respectively. In the PARADIGM study published by Haddad et al., patients in ICT arm received 3 cycles of TPF plus CRT with weekly carboplatin, 67% of patients were progress free and 73% were alive in 3 years after treatment [4]. Takacsi-Nagy et al. in phase II clinical trial involving 66 LA-SCCHN patients demonstrated that after 2 cycles of TPF followed by CRT (total dose of 70 Gy in 7 weeks with 3 concurrent cycles of cisplatin on days 1, 22 and 43 of radiotherapy) 3-year PFS rate was 41% and OS rate - 43% [27]. Ghi et al. analysed 208 patients randomised for either CRT with cisplatin (n = 129) or radiotherapy with cetuximab (n = 79) after initial 3 cycles of TPF [28]. 3-year PFS and OS rates were 47% and 57.5% respectively. In our study, involving 47 patients treated with 3 cycles of TPF plus CRT (70 Gy in 7 weeks with weekly cisplatin 40 mg/m2), we demonstrated 3-year PFS rates of 67% and 3-year OS rates of 61%. Summarizing the data, 3-year PFS and OS rates in our study were non-inferior comparing to survival results in studies with the standard treatment approach.

According to the survival data presented above, we suggest that 18F-FDG PET/CT scan obtained after TPF-based ICT might be used for LA-SCCHN IMRT planning. Loco-regional failure analysis also supports this approach. In our study 15 patients (31.9%) developed loco-regional disease progression with a total of 32 progressive lesions. 8 lesions were in the primary tumor site and 24 in the regional lymphnode sites. 90.6% of progresses were in-field with highest levels localized in high-to-intermediate risk volumes GTV70 (87.5%) and CTV60 (87.5%) and none in PTV50 volume. For comparison, De Felice et al. presented the retrospective analysis of 56 patients who were diagnosed with loco-regional progress of LA-SCCHN after ICT plus CRT [23]. In total 68 sites of progression were analyzed, of them 35 were in primary tumor site and 33 in regional nodes. 95.6% of progressive lesions were in-field, 82.9% in high-dose primary tumor CTV (GTV + 10 mm), and 72.7% in nodal high-dose CTV. Similar results were demonstrated by Bayman et al. [29]. This study involved 136 patients with carcinoma of the head and neck. 29 (21%) patients were treated with ICT plus CRT. 16 (12%) of patients progressed, all in high-dose regions. The main difference between the studies mentioned above and our study is GTV to CTV margin. In both earlier studies CTV was defined as GTV plus 10 mm margin and PTV–CTV plus 4 mm. In our study smaller 5 mm GTV to CTV margin and 3 mm CTV to PTV margin (accounting for daily CBCT image guidance) was used. We want to point-out that despite the smaller margins, results of loco-regional control were similar to previous studies, therefore we suggest that 5 mm CTV and 3 mm PTV margins (with daily CBCT image guidance) might be considered for ^18^F-FDG PET/CT based IMRT planning after ICT for LA-SCCHN patients.

In recent years several authors demonstrated a prognostic value of ^18^F-FDG PET/CT metabolic parameters in LA-SCCHN [15,16,30,31,32]. In publication by Paidpally et al. SUV_max_, MTV and TLG were proposed as non-invasive prognostic factors usable in management of LA-SCCHN [30]. These parameters might be used for disease response evaluation, RT planning and follow-up. David et al. analyzed 74 LA-SCCHN patients with N2 or N3 nodal status [15]. SUV_max_, SUV_peak_ (mean SUV within a 1-cm sphere centered on SUV_max_) of the primary tumor and LN were evaluated before and after treatment. Neither initial nor post-treatment SUV_max_ and SUV_peak_ were associated with disease outcome. Choosing the different approach and analyzing the dynamics of SUV_max_ (percentage decrease of the value after ICT) on patient survival, we provide new evidence that the decrease of primary tumor SUV_max_ by at least 74% and LN SUV_max_ by 68–69% may be used as independent LA-SCCHN prognostic factors. However, our results contradict the results of Yu et al., who did not find an association between SUV_max_ percentage decrease and LA-SCCHN patient survival [16].

Few authors also investigated the prognostic value of MTV and TLG. In the study of David et al., pre-treatment MTV value was linked with PFS and OS [15]. Yu et al. found that post-ICT MTV percentage decrease by more than 42% and TLG by more than 55% significantly prolonged event-free survival [16]. In our study it was found that larger percentage decrease of MTV and TLG in primary tumor and TLG in regional LN correlate with better 3-year PFS and OS rates, however different cut-off points were established. MTV percentage decrease cut-off point for PFS prediction was 68%, for OS 69%. Only primary tumor MTV decrease was significantly associated with disease outcome. As for TLG better PFS was observed in patients with ≥76% primary tumor TLG decrease and ≥74% nodal TLG decrease with similar results for OS ≥74% and ≥73% decrease respectively.

Limitations of this study include the usage of rigid image registration technique, which was used for IMRT treatment planning and evaluation of recurrence patterns. There are no clear recommendations in the literature regarding which image registration technique should be used for LA-SCHHN IMRT planning. However, deformable registration seems to be a superior technique. Another limitation was the lack of endoscopic tumor evaluation after ICT (21 out of 47 patients refused the second examination). Due to this limitation, comparison of post-ICT residual primary tumor between PET/CT with objective endoscopic evaluation method was not possible.

## 5. Conclusions

Results of this phase II cohort study demonstrate that post-ICT ^18^F-FDG PET/CT based RT planning has no negative impact on loco-regional recurrence rates comparing with the patterns to pre-ICT imaging-based treatment planning (demonstrated by others authors). Furthermore, we demonstrated that SUV_max,_ MTV and TLG percentage decreases in primary tumors, and LN TLG decreases are independent prognostic factors for PFS and OS in LA-SCCHN. In order to improve the analysis of loco-regional progression and assess the future possibility of dose escalation in high-risk volumes, we are initiating a 3D-printed phantom prototype filled with radiosensitive gels-based dosimetry for individual patient dosimetry according to RT plans. Further prospective studies with larger sample sizes are warranted to confirm the ^18^F-FDG-PET/CT based post-ICT target volume delineation technique before its use in clinical practice. Also, we encourage larger validation studies of metabolic ^18^F-FDG-PET/CT prognostic markers.

## Figures and Tables

**Table 1:** Baseline patient and tumor characteristics.

Characteristics	Value
Age, median (range), years	55.5 (30–71)
Sex, *n* (%)	
Male	45 (95.7)
Female	2 (4.3)
ECOG, *n* (%)	
0	30 (63.8)
1	17 (36.2)
Primary tumor site, *n* (%)	
Oropharynx	26 (55.3)
Hypopharynx	21 (44.7)
Tumor status (T), *n* (%)	
T1	0
T2	14 (29.8)
T3	9 (19.1)
T4	24 (51.1)
Lymph node status (N), *n* (%)	
N0	3 (6.4)
N1	9 (19.1)
N2	32 (68.1)
N3	3 (6.4)
Tumor stage, *n* (%)	
III	7 (14.9)
IV	40 (85.1)
Histological grade, *n* (%)	
G1	1 (2.1)
G2	27 (57.4)
G3	19 (40.5)
G4	0

ECOG—Eastern Cooperative Oncology Group performance status.

**Table 2:** Sites of loco-regional failures and RT doses in post-ICT PET/CT based IMRT plans.

Characteristic	Failures, n (%)	Dose, Average ± SD			
Type		Mean Dose	Minimum Dose	Maximum Dose	D95
Primary site					
In-field	5 (62.5)	67 ± 4.3	65 ± 3.5	71 ± 2.9	67.64 ± 2.6
Marginal	1 (12.5)	69.9	64.41	71.7	68.4
Out-field	2 (25.0)	58.5 ± 14.85	49.5 ± 21	71.7 ± 0.1	51.71 ± 24
	*p* value	0.41	0.25	0.9	0.2
LN					
In-field	14 (58.3)	64.4 ± 3.15	61.93 ± 3.40	67.03 ± 2.96	62.8 ± 3.40
Marginal	7 (29.2)	62.3 ± 1.49	55.8 ± 9.60	66 ± 2.65	59.6 ± 8.36
Out-field	3 (12.5)	59.4 ± 7.36	59.47 ± 7.40	68.1 ± 2.98	58.9 ± 8.13
	*p* value	0.14	0.08	0.8	0.37
Primary site					
GTV *	5 (62.5)	66.9 ± 4.26	65 ± 3.50	71 ± 2.90	67.64 ± 2.60
CTV *	2 (25.0)	69.44 ± 0.60	64.3 ± 0.15	71.84 ± 0.23	68.5 ± 0.24
PTV *	1 (12.5)	48	34.7	71.7	34.7
	*p* value	0.013	0.001	0.8	0.001
LN					
GTV **	14 (58.3)	64.4 ± 3.15	61.93 ± 3.40	67.03 ± 2.96	62.8 ± 3.40
CTV **	6 (25.0)	61.84 ± 0.90	54.92 ± 10.20	65.93 ± 2.90	59 ± 9.01
PTV **	4 (16.7)	60.9 ± 6.64	56.6 ± 8.70	67.65 ± 2.60	59.9 ± 6.94
	*p* value	0.15	0.08	0.91	0.37

* high-dose (70 Gy) GTV, CTV and PTV of primary site failures; ** intermediate-dose (60 Gy) GTV, CTV and PTV of lymph nodes failures; LN—regional lymph nodes.

**Table 3:** Sites of loco-regional failures and RT doses in pre-ICT PET/CT based IMRT plans.

Characteristic	Failures, n (%)	Doses Average (± SD)			
Type		Mean Dose	Minimum Dose	Maximum Dose	D95
Primary site					
In-field	6 (75)	67.2 ± 3.9	64.8 ± 3.4	71.1 ± 2.6	66.3 ± 4.6
Marginal	2 (25)	63.7 ± 8.9	60.1 ± 6.2	71.2 ± 0.6	68 ± 0.1
Out-field	-	-	-	-	-
	*p* value	0.42	0.18	0.9	0.17
LN					
In-field	18 (75)	63.7 ± 3.1	59.7 ± 7.1	66.8 ± 3.1	61.3 ± 5.9
Marginal	4 (16.7)	61.8 ± 6.1	56.7 ± 8.6	67.3 ± 2.8	63.3 ± 7.1
Out-field	2 (8.3)	64.1 ± 1.5	60.9 ± 0.2	66.35 ± 0.5	63.9 ± 1.4
	*p* value	0.217	0.69	0.92	0.76
Primary site					
GTV *	6 (75)	67.2 ± 3.9	64.8 ± 3.1	71.1 ± 2.6	67.8 ± 2.3
CTV *	2 (25)	63.6 ± 8.9	60.1 ± 6.2	71.2 ± 0.6	64.4 ± 8.4
PTV *	-	-	-	-	-
	*p* value	0.42	0.18	0.76	0.17
LN					
GTV **	18 (75)	63.97 ± 2.9	61.6 ± 3.1	66.7 ± 2.8	63.03 ± 3
CTV **	3(12.5)	60.6 ± 6.9	52.3 ± 8.7	67.35 ± 3.8	56.62 ± 6.3
PTV **	3 (12.5)	63.03 ± 2.1	52.4 ± 11.8	68.1 ± 3.0	54.53 ± 12.8
	*p* value	0.07	0.013	0.67	0.04

* high-dose (70 Gy) GTV, CTV and PTV of primary site failures; ** intermediate-dose (60 Gy) GTV, CTV and PTV of lymph nodes failures; LN—regional lymph nodes.

**Table 4:** AUC and cut-off values of metabolic parameters.

Parameter			PFS			OS		
			AUC	Optimal Cut-Off, %	Sensitivity/Specificity, %	AUC	Optimal Cut-Off, %	Sensitivity/Specificity, %
Primary tumor	percentage decrease of:	SUV_max_	0.79	74	68/88	0.70	74	69/67
		MTV	0.83	68	71/88	0.76	69	76/78
		TLG	0.78	76	74/88	0.78	74	74/81
Metastatic LN	percentage decrease of:	SUV_max_	0.72	68	68/89	0.68	69	69/67
		MTV	0.58	-	-	0.50	-	-
		TLG	0.79	74	62/87	0.71	73	72/69

AUC—area under the curve; LN—regional lymphnodes; PFS—progression-free survival; OS—overall survival; “-“—no optimal cut off point.

**Table 5:** Associations of ^18^F-FDG PET/CT metabolic parameters with tumor response to ICT in primary tumor.

Parameter of the Primary Tumor		Total Mean ± SD	ICT-Responders Mean ± SD	ICT-Non-Responders Mean ± SD	*p* Value
Pre-ICT	SUV_max_	17.9 ± 5.8	17.6 ± 1.1	18.9 ± 1.4	0.910
	MTV	34.6 ± 26.6	27.7 ± 3.3	52.7 ± 9.7	<0.001
	TLG	232.3 ± 209.2	178 ± 24.1	372 ± 77.5	0.020
Post-ICT	SUV_max_	7.2 ± 5.02	3.1 ± 0.9	5.9 ± 3.5	0.004
	MTV	11.8 ± 15.7	6.04 ± 6.9	27.04 ± 21.7	<0.001
	TLG	60.05 ± 91.11	27.2 ± 56.2	146 ± 110	<0.001
Percentage decrease (%)	SUV_max_	58.9 ± 24.7	68.3 ± 2.7	34.6 ± 7.2	0.027
	MTV	63.4 ± 33.6	74.4 ± 3.2	34.7 ± 12.2	0.001
	TLG	66.3 ± 41.2	68.3 ± 2.7	34.6 ± 7.2	0.950

SUV_max_—maximum standard uptake value; MTV—metabolic tumor volume; TLG—total lesion glycolysis; SD—standard deviation; ICT—induction chemotherapy.

**Table 6:** Associations of ^18^F-FDG PET/CT metabolic parameters with tumor response to ICT in metastatic LN.

Parameter of the Metastatic LN		Total Mean ± SD	ICT-Responders Mean ± SD	ICT-Non-Responders Mean ± SD	*p* Value
Pre-ICT	SUV_max_	13.02 ± 6.4	12.4 ± 1.1	14.9 ± 1.9	0.820
	MTV	19.3 ± 28.2	12.1 ± 2.4	40.9 ± 13	<0.001
	TLG	140.3 ± 234.9	77.1 ± 15	330.2 ± 109.7	<0.001
Post-ICT	SUV_max_	7.5 ± 5.7	3.3 ± 1.8	5.5 ± 2.5	0.115
	MTV	50.5 ± 129.2	24.1 ± 58.5	129.54 ± 227.2	0.006
	TLG	60.05 ± 91.11	27.2 ± 56.2	146 ± 110	<0.001
Percentage decrease (%)	SUV_max_	38.5 ± 32.8	44 ± 5.7	22.2 ± 7.3	0.340
	MTV	38.5 ± 32.8	62 ± 4.7	39.1 ± 12.2	0.211
	TLG	64.9 ± 34.6	72.3 ± 4.3	42.9 ± 13.4	0.090

SUV_max_—maximum standard uptake value; MTV—metabolic tumor volume; TLG—total lesion glycolysis; SD—standard deviation; LN—regional lymphnodes.

**Table 7:** Univariate and multivariate Cox proportional hazards regression analysis for PFS.

Parameter		Cut-Off Value	Univariate			Multivariate		
			HR	95%CI	*p* Value	HR	95%CI	*p* Value
Primary tumor percentage decrease of:	SUV_max_	<74 vs. ≥74%	1.3	1.1–2.1	0.04	1.4	1.2–9.3	0.04
	MTV	<68 vs. ≥68%	1.5	1.3–9.5	0.01	1.9	1.3–11.5	0.03
	TLG	<76 vs. ≥76%	4.9	1.8–13.6	0.002	3.7	1.2–9.3	0.03
Metastatic LN percentage decrease of:	SUV_max_	<68 vs. ≥68%	1.2	1.2–2.2	0.04	0.7	0.2–2.7	NS
	MTV	*	-	-	-	-	-	-
	TLG	<74 vs. ≥74%	1.6	1.5–4.2	0.03	2.9	1.3–6.9	0.03
Tumor status (T)		T2-3 vs. T4	0.9	0.5–2.3	NS	-	-	-
Lymphnode status (N)		N0-1 vs. N2-3	3.4	1.6–7.8	0.01	2.8	1.7–16.3	0.02
Tumor site		Oropharynx vs. hypopharynx	0.7	0.3–1.7	NS	-	-	-
Histological grade		G1-2 vs. G3	0.3	0.24–1.4	NS	-	-	-

SUV_max_—maximum standard uptake value; MTV—metabolic tumor volume; TLG—total lesion glycolysis; LN—regional lymphnodes; * no optimal cut-off value; NS—not significant.

**Table 8:** Univariate and multivariate Cox proportional hazards regression analysis for OS.

Parameter		Cut-Off Value	Univariate			Multivariate		
			HR	95%CI	*p* Value	HR	95%CI	*p* Value
Primary tumor percentage decrease of:	SUV_max_	<74 vs. ≥74%	2.5	1.3–7.9	0.03	1.2	1.1–5.8	0.04
	MTV	<69 vs. ≥69%	1.8	1.3–5.4	0.04	1.8	1.3–6.7	0.03
	TLG	<74 vs. ≥74%	3.2	1.3–7.7	0.01	3.1	1.4–17.6	0.02
Metastatic LN percentage decrease of:	SUV_max_	<69 vs. ≥69%	1.4	1.2–5.5	0.03	0.8	0.7–2.3	NS
	MTV	*	-	-	-	-	-	-
	TLG	<73 vs. ≥73%	2.2	1.4–11.5	0.02	2.6	1.4–6.2	0.02
Tumor status (T)		T2-3 vs. T4	0.8	0.6–3.4	NS	-	-	-
Lymphnode status (N)		N0-1 vs. N2-3	3.9	1.3–9.4	0.03	2.2	1.4–8.7	0.02
Tumor site		Oropharynx vs. hypopharynx	0.7	0.3–1.7	NS	-	-	-
Histological grade		G1-2 vs. G3	0.6	0.2–1.4	NS	-	-	-

SUVmax—maximum standard uptake value; MTV—metabolic tumor volume; TLG—total lesion glycolysis; LN—regional lymphnodes; *—no optimal cut-off value.

**Table 9:** Patient survival data in previous studies with ICT plus CRT approach and in our study.

Study	Eligibility	Patients	Study Design	3-Year OS Rates	3-Year PFS Rates
Lorch et al. [26]	Stage III-IV	501	TPFx3, SRT+CBP weekly vs. CP/5-Fu, SRT+CBP weekly	62%	50%
Haddad et al. [4]	Stage III-IV	145	TPFx3, SRT+CBP weekly vs. HRT+CPx2	73%	67%
Takacsi-Nagy et al. [27]	Stage III-IV	66	TPFx2, SRT+CPx3 vs. CRT	43%	41%
Ghi et al. [28]	Stage III-IV	413	TPFx3, SRT+CPx2 vs. SRT+CTX	57.5%	47%
Our study	Stage III-IV	47	TPFx3, SRT+CP weekly	61%	67%

TPF: docetaxel, cisplatin, 5-Fluorouracil; SRT: single daily fraction radiotherapy; HRT: hyperfractionated radiotherapy; CBP: carboplatin; CP: cisplatin: CTX: cetuximab; CRT: chemoradiotherapy; 5-FU: 5-fluorouracil.
